# Shaken batch cultures of Pseudomonas aeruginosa contain biofilm-like heterogeneity

**DOI:** 10.1099/mic.0.001696

**Published:** 2026-06-04

**Authors:** B. G Fritz, M.H Christensen, L Heiland, T Tolker-Nielsen, T.H Jakobsen, PØ Jensen, T Bjarnsholt

**Affiliations:** 1 Department of Immunology and Microbiology, University of Copenhagen, Copenhagen, Denmark; 2 Department of Clinical Microbiology, Rigshospitalet, Copenhagen, Denmark; 3 Deparment of Bacteriology and Immunology, University of Helsinki, Helsinki, Finland; 4 Institute for Inflammation Research, Center for Rheumatology and Spine Diseases, Rigshospitalet, Copenhagen, Denmark

**Keywords:** aggregation, antibiotic tolerance, biofilm, growth rate, heterogeneity, *Pseudomonas*

## Abstract

Two potential sources of differentially tolerant subpopulations of *Pseudomonas aeruginosa* in batch cultures are aggregation and growth rates. Here, we dissected these effects by treating batch cultures with tobramycin, colistin and a combination after various time intervals. Tobramycin was significantly more effective than colistin at early time points, while both showed similar efficacy at 24 and 48 h. Combination therapy was most effective at later stages, indicating the emergence of tolerant subpopulations. Reactivating growth of stationary-phase cultures by treating in fresh media altered antibiotic tolerance, further supporting the role of growth rate in tolerance. To examine the effect of aggregation, we separated batch cultures using filtration as well as examined the WT against a hyper-aggregating ΔwspF mutant. Aggregates demonstrated lower growth rates than single cells. However, microcalorimetry and colony enumeration after treatment revealed that aggregation only partially explained the variance associated with antibiotic treatment. Given that tobramycin and colistin target metabolically distinct subpopulations, this suggests that overall metabolic rate plays a larger role than aggregation regarding antibiotic tolerance in batch cultures.

## Data Availability

Data and code to reproduce the figures and analysis in this manuscript can be found at https://github.com/bgfritz1/Pa_Subpopulations. Additional raw data will be provided by the co-authors upon request.

## Introduction

Bacteria exist either as single cells or as aggregations of single cells, known as ‘biofilms’. Bacterial biofilms are found in the environment, as well as in and on the human body [1]. Costerton *et al*. define biofilm as ‘matrix-enclosed bacterial populations adherent to each other and/or to surfaces or interfaces’ [2]. Most laboratory models for studying biofilms rely on the attachment and growth of a bacterial biofilm on a solid surface, which is easily accomplished with *in vitro* models. These models include the CDC Biofilm Reactor^®^ [3], the drip-flow reactor [4,5], multi-well plate methods [6,7], flow-cell systems [8] and others [9]. These traditional models of biofilm formation involve the attachment of cells to a surface, leading to a subsequent phenotypic shift towards the biofilm phenotype and development of the biofilm [10]. Once established, these biofilms develop chemical, nutritional and signalling gradients, which generate distinct internal microenvironments, containing differentiated subpopulations of cells with distinct physiology [11]. For instance, cells within the mushroom structures of *Pseudomonas aeruginosa* biofilms in a flow cell were shown to be less metabolically active and, thus, less susceptible to certain antibiotics, such as tobramycin, while outer cells were more active and susceptible [12]. The relevance of these model systems, especially for replicating *in vivo* biofilms, is a topic of continued discussion [13,15].

The biofilm phenotype *in vivo* is commonly associated with chronic and persistent infections. Common examples of this are chronic bacterial pneumonia in persons with cystic fibrosis, chronic non-healing wounds and implant-associated infections [16,18]. The biofilms in these infections may exhibit similarities to the biofilms grown *in vitro*, such as increased tolerance to antibiotic challenges [19]. It is commonly presumed that the increase in antibiotic tolerance of chronic infections is due to the formation of bacterial biofilms, as observed *in vitro*. However, the biofilms observed *in vivo* show a fundamentally different structure from biofilms observed in the *in vitro* model systems. *In vivo* biofilms generally consist of small aggregates, which are not necessarily attached to a solid surface [13,20]. These observations contradict the assumption that surface attachment and/or aggregation is necessary to bring about biofilm-like properties [15]. Previous studies have identified that several common laboratory strains form non-surface-attached aggregates in both stationary and shaken batch cultures [21,23]. Non-surface-attached aggregates in non-shaken batch cultures appeared to exhibit similar phenotypic traits as surface-attached biofilms, such as increased tolerance towards antibiotics, which decreased when aggregates were mechanically disrupted and treated with tobramycin in fresh media [21]. It is unclear whether this increased tolerance is a consequence of aggregation or simply a result of the decreased metabolism caused by restricted access to nutrients. Some planktonic bacterial cells under stress also demonstrate biofilm-like traits, including slow metabolism and increased tolerance to antibiotics, as demonstrated by so-called ‘persister cells’ and viable-but-non-culturable cells (VBNCs) [24,26]. The differentiation, if any, between persister cells, VBNC, slow-growing biofilm and stationary-phase planktonic cells is unclear and probably represents the same phenotype. Here, we raise the question – could the increased antibiotic tolerance and other biofilm-like properties be due to the growth rate and physiological status of individual bacterial cells, rather than a response caused by biofilm formation or surface attachment?

This study utilized treatment with tobramycin and colistin to investigate differentially tolerant and metabolically distinct subpopulations in batch cultures of *P. aeruginosa* containing both single cells and aggregates. Tobramycin and colistin act most strongly upon metabolically active and inactive cells, respectively [27,29]. Liquid batch cultures were treated with these antibiotics after 2, 4, 8, 24 and 48 h to identify when tolerant subpopulations arise. In addition, the effect of growth rate on the development of these subpopulations was examined by treating 24 h cultures in either spent or fresh media. We hypothesized that treatment in fresh media would revive a slow-growing population and increase susceptibility to tobramycin. We also hypothesized that biofilm-like properties, such as increased antibiotic tolerance, are related to the individual bacteria’s physiological state rather than the formation of a biofilm, i.e. slow-growing but planktonic bacteria may exhibit similar antibiotic tolerances as bacteria in aggregates or in biofilms. To examine this, we compared the bacterial activity of single and aggregated cells by measuring the fluorescence of a transformed *P. aeruginosa* strain with an unstable green-fluorescence protein, whose expression was induced by an arabinose-inducible promoter [30].

## Methods

### Organisms and culture conditions

*P. aeruginosa* PAO1 ATCC 15692 or an isogenic Δ*wspF* mutant [31] was used in all antibiotic tolerance experiments. A *P. aeruginosa* PAO1 wild-type strain carrying a miniTn5-Gm transposon of an araC-P_BAD_-gfp cassette was used for the fluorescence intensity experiments and provided by Morten Hentzer [30]. A 5 ml volume of lysogeny broth (LB) in a 15 ml culture tube was inoculated from frozen bacterial stock with a 1 µl inoculating loop and incubated overnight. ABTG (A-10 buffer + B-trace minimal media supplemented with 5% glucose) was prepared by mixing 900 mL of B-trace media (200 mg L⁻¹ CaSO₄·2H₂O, 200 mg L⁻¹ FeSO₄·7H₂O, 20 mg L⁻¹ MnSO₄·H₂O, 20 mg L⁻¹ CuSO₄·5H₂O, 20 mg L⁻¹ ZnSO₄·7H₂O, 10 mg L⁻¹ CoSO₄·7H₂O, 12 mg L⁻¹ NaMoO₄·H₂O, and 5 mg L⁻¹ H₃BO₃), 75mL of A-10 buffer (30 g L⁻¹ NaCl, 30 g L⁻¹ KH₂PO₄, 60 g L⁻¹ Na₂HPO₄·2H₂O, and 20 g L⁻¹ (NH₄)₂SO₄), and 5 mL of 10% glucose. For antibiotic tolerance experiments, a 250 ml Erlenmeyer flask containing 100 ml of ABTG media was inoculated with 1 ml from this overnight culture. This regrowth culture was then grown to OD_450_=0.5 before being used in treatment experiments. All cultures were incubated at 37 °C on a shaking table set to 180 r.p.m., unless otherwise stated.

### Antibiotic stock preparation

To make the tobramycin stock, 5 ml of milli-Q ultrapure H_2_O (MQ) was added 700 mg tobramycin sulphate (~500 mg tobramycin) (Meda, Sweden). The solution was stored at 4 °C until used. The final concentration of tobramycin stock is 100 mg ml^−1^. To make the colistimethate sodium stock, 1.6 ml of MQ H_2_O was added to 80 mg colistimethate sodium (Profile Pharma LTD, Chichester, Great Britain). The solution was stored at 4 °C. The final stock concentration of colistin is 50 mg ml^−1^. For colistin sulphate (Sigma-Aldrich, St. Louis, USA), 1–2 mg of colistin sulphate powder was weighed and dissolved in a volume of MQ and diluted to a concentration of 1 mg ml^−1^.

### Growth phase and antibiotic tolerance

Three 250 ml Erlenmeyer flasks containing 100 ml of ABTG media were each inoculated with 1 ml from the regrowth culture to obtain an initial OD_450_ of 0.005. At each treatment time point (2, 4, 8, 24 and 48 h after inoculation), 6 ml from each flask were transferred into an empty and sterile 50 ml conical vial using a serological pipette. Antibiotics were applied by transferring 1.9 ml aliquots from the conical vial into 15 ml culture tubes containing either a 100 µl volume of treatment (2 mg ml^−1^ tobramycin, 0.5 mg ml^−1^ colistin or MQ H_2_O control) or 200 µL volume of combination treatment (100 µL, 2 mg ml^−1^ tobramycin+100 µl, 0.5 mg ml^−1^ colistin). The final treatment concentrations of antibiotics in the tubes after addition of the sample were 100 µg ml^−1^ tobramycin or 25 µg ml^−1^ colistin, either individually or combined. These concentrations correspond to ~100× MIC and were chosen due to previously observed findings of high colistin/tobramycin tolerance in biofilms as well as to lead to killing rather than inhibition [12]. Dilution of the sample by antibiotic was ignored, given the relatively small volume of treatment added. This experiment was repeated a total of four times on different days.

### Antibiotic tolerance in fresh vs used media

Three 250 ml Erlenmeyer flasks containing 100 ml of ABTG media were each inoculated with 1 ml from a regrowth culture and incubated for 24 h. To prepare the ‘used’ media, two of these three cultures were aliquoted into 50 ml conical vials (50 ml per vial). The vials were then centrifuged in a microcentrifuge for 10 min at 7,500 ***g***. The supernatants were combined and filtered through a 0.22 µm filter using a 50 ml syringe. Twenty-nine millilitre aliquots from the filtered solution were added to 4×50 ml Erlenmeyer flasks. Four additional flasks were filled with 29 ml of fresh ABTG media. Antibiotics were introduced to the flasks by pipetting 100 µl of the antibiotic stock cultures into their respective flask. The combination treatment flasks received 100 µl of each, tobramycin and colistin. The final antibiotic concentrations were 10 µg ml^−1^ tobramycin and 2.5 µg ml^−1^ colistin. Concentrations were reduced to ~10 x MIC for these experiments due to the lower number of challenge organisms. One millilitre of 24 h culture was pipetted into each flask containing the treatment. The flasks were incubated for an additional 24 h. This experiment was repeated five times on different days.

### Sampling and plating

Treated samples were processed immediately following the 24-h incubation with antibiotics. For sampling, 1 ml was collected from each treated sample and placed in a 1.5 ml microcentrifuge tube. The tubes were then centrifuged for 5 min at 6,000 ***g***. The supernatant was discarded, and the pellet was resuspended in 1 ml MQ H_2_O. This washing step was then repeated. The samples were then spun down a final time and resuspended in 1 ml PBS. The tubes were then sonicated in a degassed, sonicating water bath for 5 min (Branson Ultrasonics, USA). A dilution series was performed by diluting 0.1 ml of the sample into 0.9 ml PBS. Samples were diluted to 10^−7^. Each dilution was drop plated in 5×10 µl drops on LB plates. Plates were incubated for 24 h at 37 °C before being enumerated.

### Activity measurements of planktonic and aggregated cells

To compare the activity of single and aggregated cells, the fluorescence of aggregates and single cells of a *P. aeruginosa* PAO1 reporter strain possessing an unstable GFP protein with a half-life of 40–120 min under the control of an arabinose-inducible promoter (see: *Organisms and culture conditions*) was measured following the addition of arabinose [30,32]. Briefly, three 250 ml Erlenmeyer flasks containing 100 ml ABTG media were inoculated with 1 ml from three overnight cultures of the reporter strain. These cultures were then inoculated into 100 ml ABTG and incubated overnight. After incubation, 0.2066 g d-arabinose was dissolved in 4 ml ABTG media in a 15 ml conical tube, one per culture. The contents of the tubes were dumped into the bacterial cultures and incubated for 45 min. One 20 ml aliquot from each culture was placed into a 50 ml conical vial and centrifuged in a fixed-angle centrifuge for 5 min at 180 ***g***. A 1 ml pipette was then used to collect the pellet and transferred to a 1.5 ml microcentrifuge tube. From each of these, a 30 µl aliquot was added to a single channel of an Ibidi µ-slide IV (Ibidi GmbH, Martinsried, Germany). The slide was then visualized on a Zeiss LSM 880. Ten fields containing at least one aggregate were selected and imaged for each sample.

### Filtration of mixed planktonic and aggregate culture

Cultures were grown as previous until an OD_450_ of 0.5 was reached. Then, 1 ml of this culture was inoculated in 100 ml of ABTG and incubated for 24 h at 37 °C (180 r.p.m.) before being separated with cell strainers (PuriSelect, Leipzig, Germany) [33].

A connector ring (PuriSelect, Leipzig, Germany) was attached to a 50 ml tube (TPP, Schaffhausen, Switzerland), followed by the 10 and 30 µm cell strainer. The culture was poured slowly through the filters, while half of the media was filter sterilized with the 0.22 µm filter (TPP, Schaffhausen, Switzerland) to create used media. The 10 µm filter was discarded and its flow-through was used for the planktonic *P. aeruginosa*. The 30 µm filter mesh was cut out with a scalpel and put into 15 ml of spent ABTG media, labelled as aggregated *P. aeruginosa*. These subpopulations were then treated and enumerated as previous experiments. Additionally, colistin sulphate was included to examine the effects against the prodrug, colistimethate sodium.

### Microcalorimetry

A *P. aeruginosa PAO1* wild-type (ATCC 15692) and a *P. aeruginosa* ΔwspF mutantwere streaked on LB agar plates (Substrate Department, Panum, University of Copenhagen) from frozen stocks and incubated overnight at 37 °C. Three colonies were picked, inoculated in 5 ml LB broth (Substrate Department, Panum, University of Copenhagen) in cell culture tubes (Greiner Bio-One, Austria) and incubated overnight at 37 °C and 200 r.p.m. orbital shaking. After incubation, the cultures were regrown by transferring 100 µl culture to 20 ml fresh LB broth in 50 ml Erlenmeyer flasks and incubated at 37 °C and 200 r.p.m. orbital shaking for 2 h to reach the exponential growth phase. Overnight cultures were used as the stationary phase cultures.

The MICs of tobramycin and colistin were determined for both the WT and wspF mutant using E-tests (bioMérieux, France). For use in the isothermal microcalorimeter (IMC), calScreener (Symcel, Sweden) antibiotic solutions were prepared and diluted in 0.9% NaCl for a final concentration of three times the observed MIC values in the well.

A total of 10 ml of the exponentially growing cultures was transferred to a 15 ml falcon tube and agitated by brief vortexing. The OD of all cultures was measured at 600 nm, and the higher ODs observed were adjusted down to match the OD of the lowest. To load the calScreener plate, 180 µl of cultures was loaded into the plastic inserts followed by 20 µl antibiotic solution. The plastic inserts were then transferred to the titanium calVials (Symcel, Sweden) and sealed with a titanium lid fitted with an O-ring. All lids were tightened to an identical torque of 40 cNm. The calPlate containing the samples was then inserted directly into the measuring chamber and allowed to run for 24 h. Data were collected at a rate of 60 Hz.

### Confocal laser scanning microscopy

Preparation of cultures was performed in the same way as for IMC. Three 10 µl drops of exponential culture from either WT or ΔwspF were placed on each slide. The drops were air dried in a LAF bench and subsequently flame fixated. The fixated bacteria were then stained with a double-labelled Texas Red PNA probe (HLB Panagene, South Korea). Slides were mounted with anti-fade mounting media and sealed with clear nail polish. An overview image was obtained with a 10 x, 0.3 NA EC Plan Neofluar objective to visualize all three drops on each slide. A tile scan grid was created consisting of five tile scans per drop. Each tile scan was 6×6 images with a size of 212.1×212.1 µm each. The centre of the tile scan grid was placed in the centre of each drop. Images were obtained with a 40×, 1.3 NA Oil DIC EC Plan Neofluar objective.

### Image analysis

Each tile scan image from every drop was analysed individually in Fiji (version 1.54 p) using a standardized workflow. The background was subtracted, and a Gaussian blur filter was applied. Brightness, contrast and thresholding were set using the built-in auto function. Particle area (in µm^2^) was measured using the ‘analyse particles’ function. Particles with an area larger than 20 µm^2^ were defined as aggregates.

### Data analysis and statistical methods

Log density (LD) was determined from the viable plate counts in the antibiotic tolerance experiments using equation 1. Mean log density (MLD) was calculated as the mean LD across biological replicates within a single experiment. Log reduction (LR) was then determined for each treatment in each experiment using equation 2.


(1) $$LD=\log_{10}\left(\frac{Mean\;CFU\;per\;drop}{0.010\;mL}\;\times Dilution\;Factor\right)$$

(2) $$LR=\;\;MLD_{Control\;}-MLD_{Treatment}$$

To determine the effect of growth phase on antibiotic tolerance, a linear regression was fitted with LR as the response and treatment and time as predictors. To determine the effect of treatment media on LR, a linear regression was fitted to LR with treatment and media (fresh or used) as predictors. Interaction effects between treatment and time or treatment and media were tested by comparing the models with their single-term deletions using the *drop1* function in R. Estimated marginal means (EMMs) were then used to estimate mean LRs for each treatment at each time or each treatment in each media, based on the fitted models, using the emmeans package [34]. Pairwise comparisons were then generated between these EMMs and reported as a compact-letter-display with the CLD function in the emmeans package. All analyses and plots from this section were generated with R statistical software [35].

Fluorescence intensities between planktonic and aggregated cells were compared by first using Fiji software to convert the raw confocal images to .tiff format. An overview image for selecting the aggregates in each confocal stack was created by summing the pixel values at each pixel position for each image in the stack. Aggregates were then manually selected using the *freehand select* tool. A mask was then created with the *mask* tool. The remainder of the analyses were then performed with a custom python script (Supplementary Materials). The pixel values in the aggregate masks were binarized, where pixels in the aggregate area had a value of 0 and pixels outside had a value of 1. A planktonic mask was then created by taking the inverse of the aggregate mask. Each .tiff image from the confocal image stack was then multiplied by the planktonic or the aggregate mask. This resulted in two confocal image stacks for each original image – one with the planktonic cells masked and one with the aggregates masked. The background fluorescence was removed from all images by setting the threshold at a fluorescence intensity value of 3,000. The difference in mean fluorescence intensity between planktonic cells and aggregated cells for an image was calculated. A mean difference for each biological replicate was calculated as the mean of the differences in fluorescence across all ten replicate images. A one-sample t-test was then used to compare whether the difference in mean was significantly different from zero.

All statistical tests were performed at a confidence level of 95%.

## Results

### Growth phase and antibiotic tolerance

**Fig. 1.**
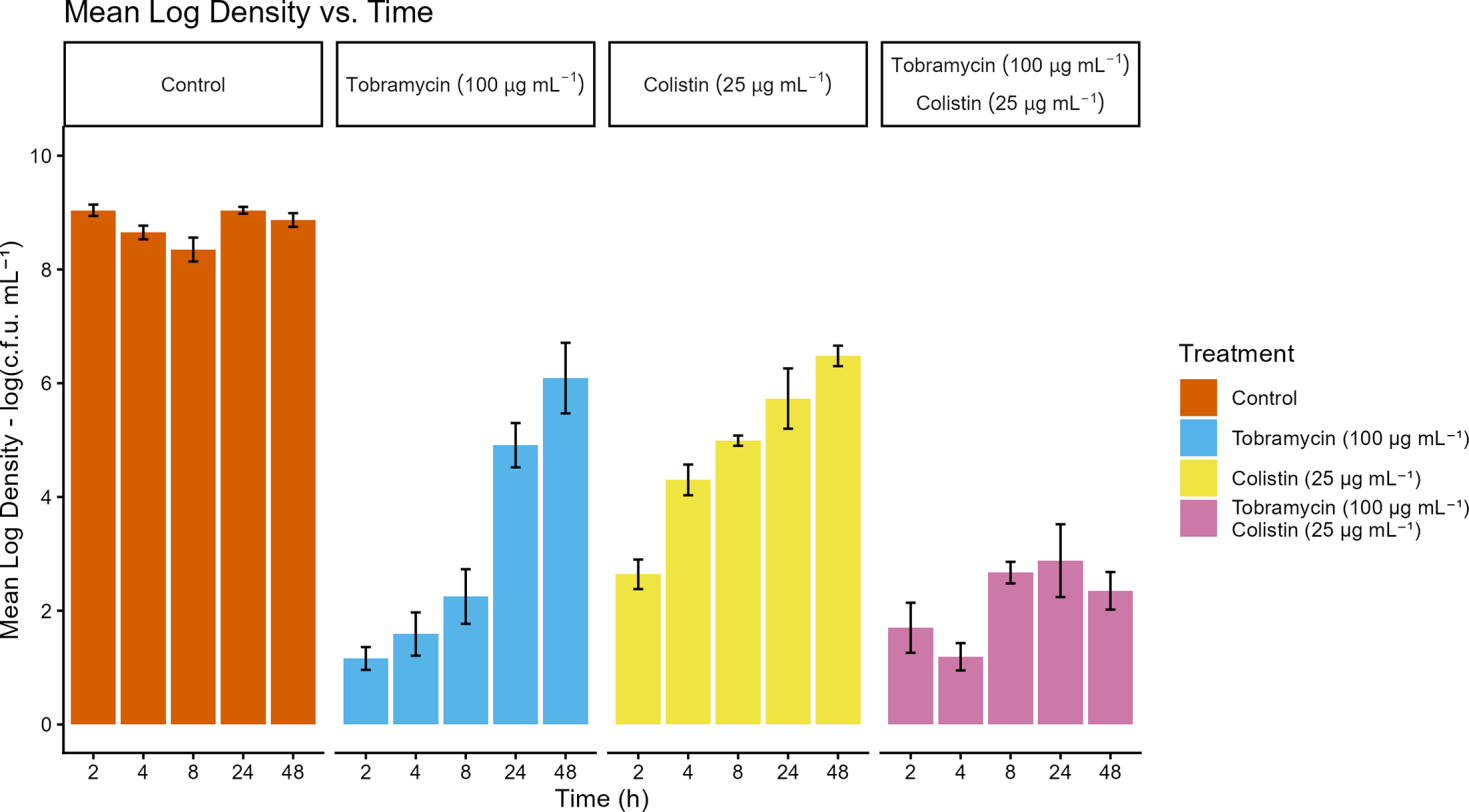
Mean log density (log_10_[c.f.u. ml^−1^]) of batch cultures grown from 2 to 48 h before treatment with 100 µg ml^−1^ tobramycin, 25 µg ml^−1^ colistin, or both antibiotics combined. Error bars represent the standard error of the mean (*n*=4).

**Fig. 2.**
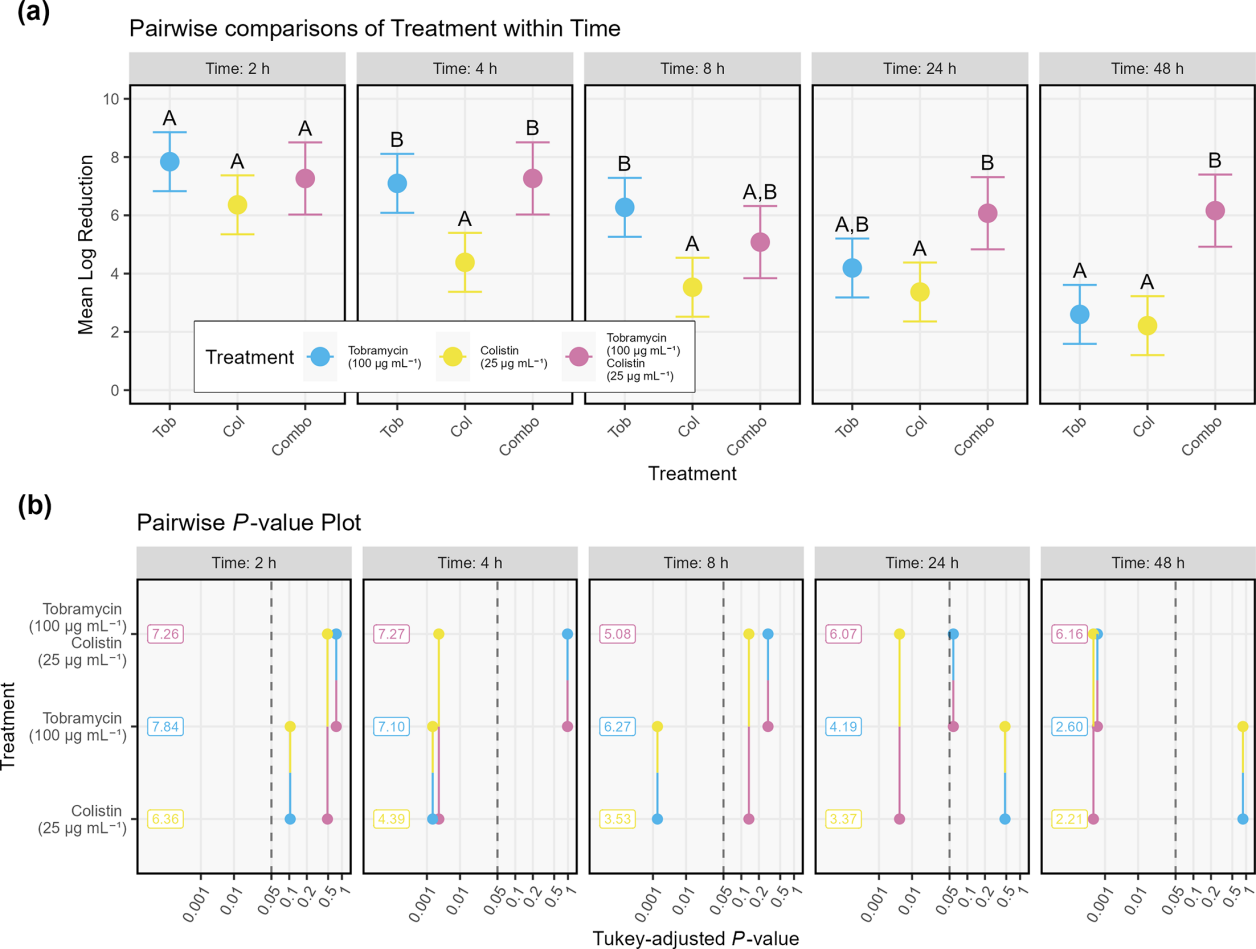
(**a**) Pairwise comparisons of mean log reductions of each treatment against the control at each time point. Error bars represent the variance estimates from the EMM model. Letters represent pairwise comparisons between conditions for each time point. If two treatments share a letter within a given time point, there is no significant difference between their mean log reductions at a significance level of 0.05. (**b**) Pairwise *P*-value plot displaying *P*-values for each pairwise comparison. Values on the left of each subplot represent the mean log reduction for that condition at a given time.

To test whether tolerance was linked to growth phase and assess the temporal development of differentially tolerant subpopulations, batch cultures were treated with tobramycin, colistin or a combination of the two after 2–48 h of growth. Mean LD values for the control and each treatment and time are displayed in Fig. 1. Mean LR values against the control and statistical groupings from the pairwise comparisons are displayed in Fig. 2(a). The LRs of colistin, tobramycin and the combination treatment decreased by 5.2, 4.1 and 1.1, respectively, from *t*=2 h to *t*=48 h. There was a significant effect of culture age on antibiotic efficacy and the effect of tobramycin and colistin single treatments decreased over time, while the effect of the combination treatment remained relatively consistent over time. The statistical significance of this effect was tested by removing the treatment:time interaction term from the regression model, which resulted in a significantly worse-fitting model (*P*=0.006). Thus, pairwise comparisons were performed separately for each time point. Comparing the treatments at each timepoint, all treatments demonstrated similar efficacy at *t*=2 h. Colistin was 2.7-fold less efficacious than tobramycin at both *t*=4 h (*P*<0.001) and *t*=8 h (*P*<0.05). The combination treatment demonstrated similar effects as tobramycin from 2 to 8 h (*P* ≥ 0.05). At 24 and 48 h, there was again no difference in the efficacies of the individual treatments. The combination treatment, however, was 3.5- and 3.9-fold more efficacious than single treatments of tobramycin and colistin at *t*=24 h and *t*=48 h, respectively. These results suggested that differentially susceptible subpopulations are present after 24 and 48 h of growth and eradicated by combination treatment.

### Antibiotic tolerance in fresh vs used media

**Fig. 3.**
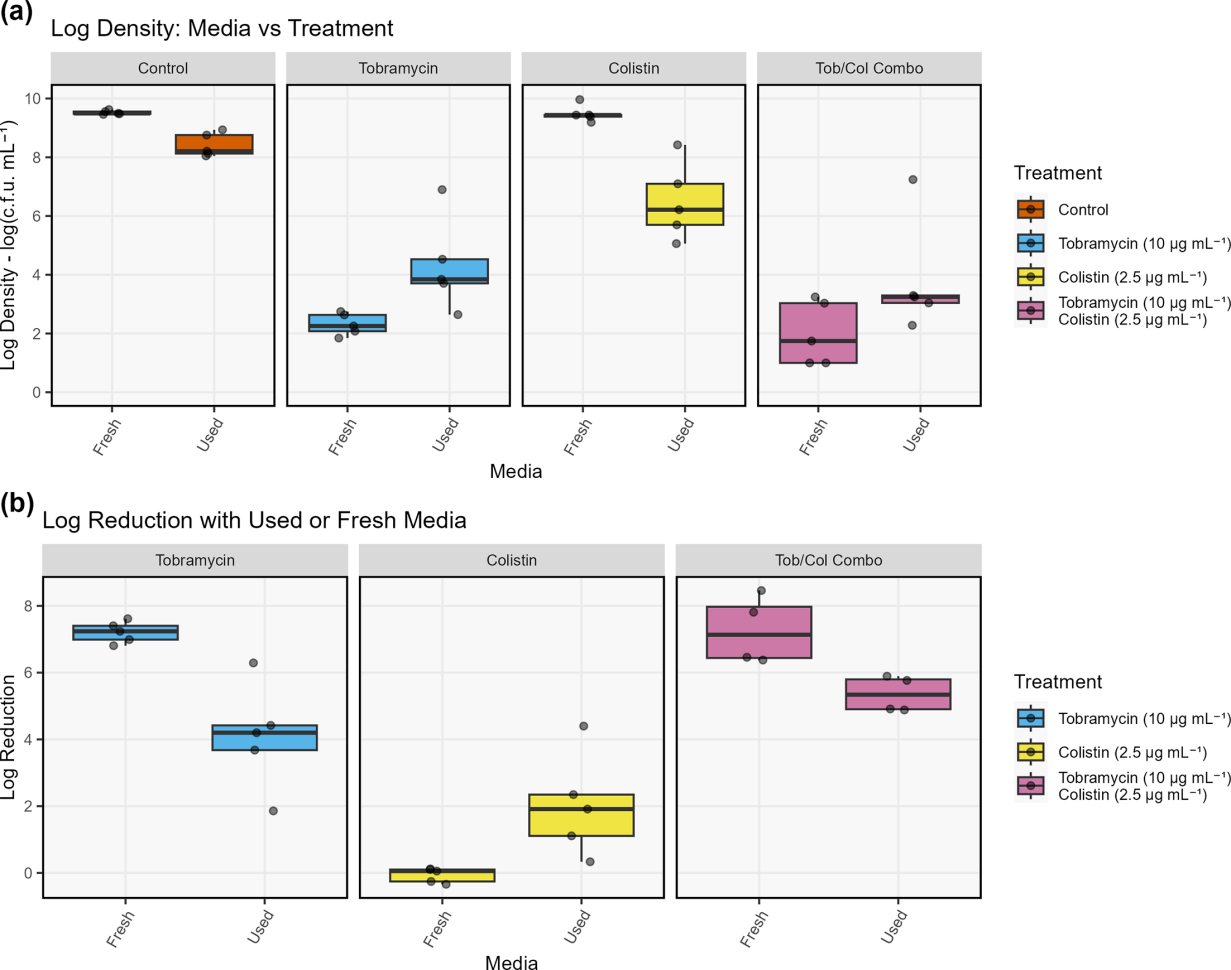
(**a**) Boxplot of log density for 24 h cultures of *P. aeruginosa* treated with tobramycin, colistin, a combination of the two and a control for 24 h in either fresh or filter-sterilized, spent media (*n*=5). (**b**) Log reduction values for each treatment against the control for the conditions described above. There was a significant effect (*P*<0.001) of media type on the log reduction for each treatment. Horizontal jitter was applied to avoid overlapping of the points.

To test whether the increased tolerance of the bacteria observed in the previous section was due to growth rate, 24 h cultures were transferred to either fresh media or sterile-filtered media from a 24 h culture grown in parallel. Mean LD and LR values for each treatment and treatment media are displayed in Fig. 3. There was a significant effect of media type on treatment efficacy for all three treatments. The LR of tobramycin increased by threefold when treatment was applied in fresh media, a significant increase relative to used media (*P*<0.001). Likewise, the LR of colistin decreased significantly (*P*<0.001) when the treatment was performed in fresh media. The combined tobramycin + colistin treatment also was significantly (*P*<0.001) more efficacious when the treatment was performed in fresh media. This suggests that the tolerant subpopulations observed previously may partially be due to different growth rates between the subpopulations.

### Fluorescence intensity of planktonic cells and aggregates

**Fig. 4.**
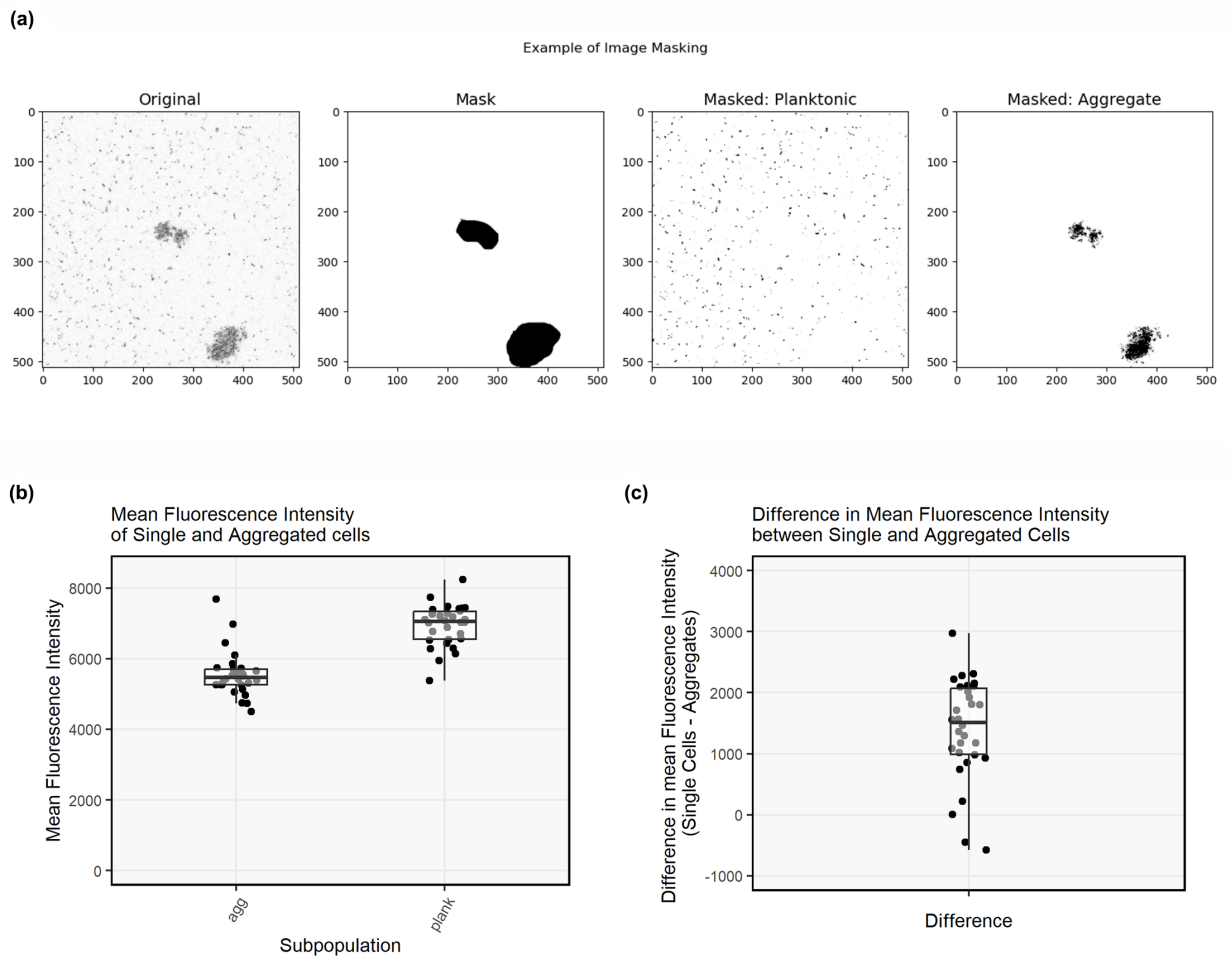
(**a**) Example of image masking procedure to isolate single and aggregated cells. The original image as a maximum intensity projection (threshold=3,000) was used to draw a mask around aggregated cells. This mask was then used to isolate either the planktonic cells (masked: planktonic) or the aggregated cells (masked: aggregates) for analysis. (**b**) Mean fluorescence intensity of pixels in the aggregated (planktonic masked) or single (aggregate masked) subpopulations. (**c**) The difference in mean fluorescence intensity between single- or aggregated cells in a given field. Points represent data from *n*=3 biological replicates, with ten random fields taken for each biological replicate.

To assess whether non-surface-attached aggregates comprise the slow-growing subpopulation, the fluorescence of planktonic and aggregated cells of *P. aeruginosa* containing an arabinose-inducible, unstable GFP reporter was measured following the addition of arabinose. Slower-growing cells with fewer ribosomes transcribe fewer copies of the unstable GFP, which has a short half-life and degrades if not continually expressed. Thus, fluorescence depends on the number of ribosomes and relates to the growth rate. Every image analysed contained both planktonic and aggregated cells. After applying a threshold cut-off of 3,000 to all images (Fig. 4a), the mean pixel intensity per image for planktonic and aggregated cells was 5,380.29 (*σ*=583.73, *n*=30) and 4,506.92 (*σ*=631.028, *n*=30), respectively, for all images across all biological replicates (Fig. 4b). The maximum pixel intensity observed for planktonic and aggregated cells was 59,445 and 54,632, respectively. The mean difference in mean fluorescence intensity among planktonic and aggregated cells was 1,397.39 (*n*=3, *σ*=443.6) (Fig. 4c), i.e. planktonic cells appeared to be brighter on average. This difference is significantly different from zero (*P*=0.03, *t*=5.45), as demonstrated by a one-sample t-test. It should be noted that this difference became more significant if the threshold was increased, decreasing the effect of background noise. This demonstrates that aggregated cells were less active than planktonic cells and produced less GFP than planktonic cells over the same timespan.

### Aggregation displays minimal effects on antibiotic efficacy and metabolism

**Fig. 5.**
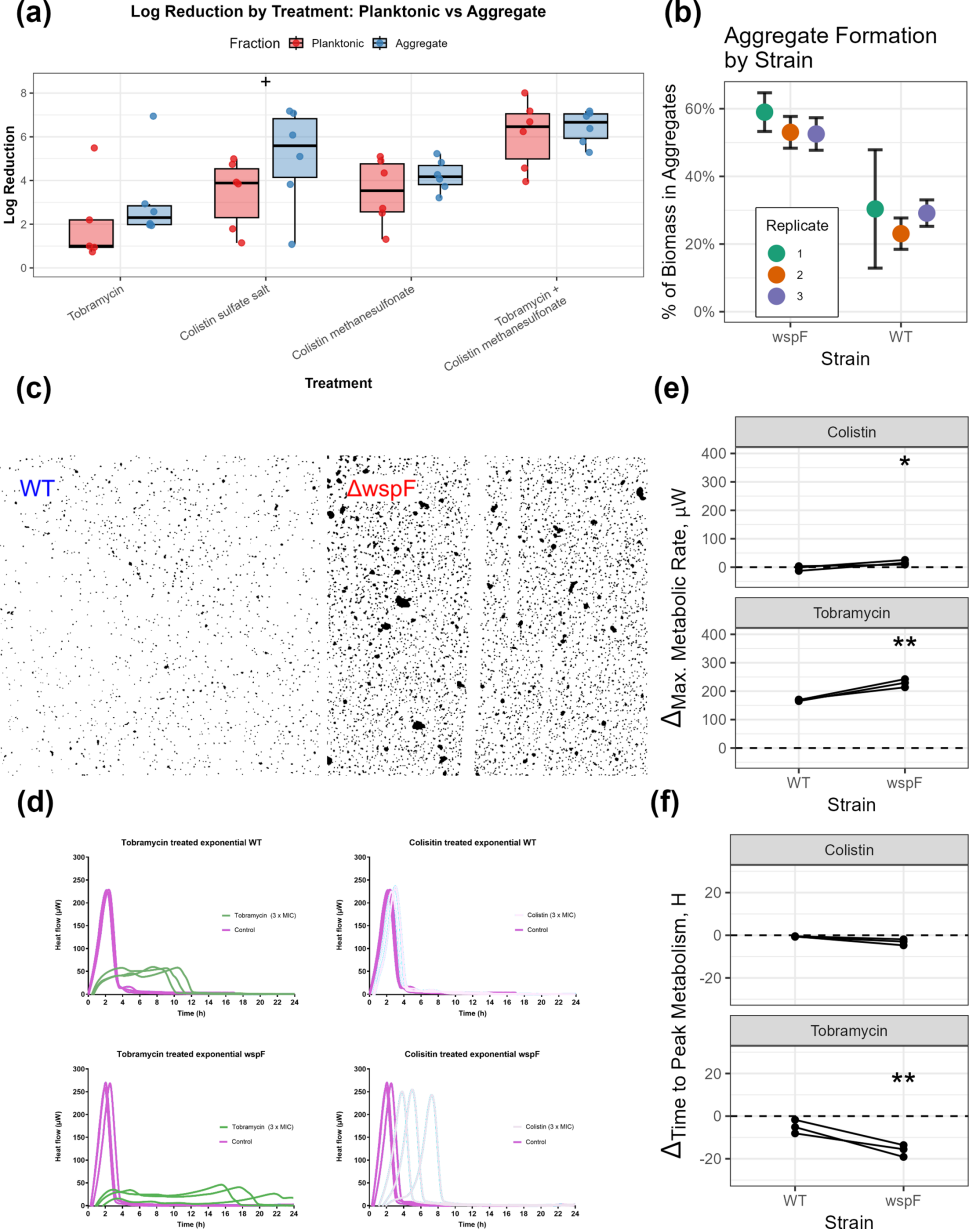
Effect of aggregation on antibiotic treatment. (**a**) Log-reduction results from treatment with tobramycin and colistin sulphate, colistimethate sodium and combined tobramycin/colistimethate on batch cultures enriched for planktonic bacteria or aggregates with filter separation. (+) indicates a marginal significance between planktonic and aggregate. (**b**) Proportion of total biomass present as aggregates for the ΔwspF and WT strains. Error bars represent the variability between technical replicates (10 µl drops with one tile-scan taken per drop, *n*=3) for each repetition of the experiment (*n*=3). (**c**) Representative fluorescent microscopy images of WT (left) and the ΔwspF mutant (left) stained with unibacterial PNA-FISH probe demonstrating increased formation of aggregates for the ΔwspF mutant. Colour images were converted to greyscale, thresholded at a pixel value of 150 and annotated. (**d**) Microcalorimetry profiles displaying heat flow (µW) over time (h) for the WT and ΔwspF mutant treated with tobramycin and colistin sulphate compared to untreated controls. (**e, f**) Effect plots demonstrating the effect of aggregation (WT vs hyper-aggregating ΔwspF) on the effect of antibiotic treatment. Antibiotic effect was quantified by mean difference (Δ) in maximum metabolic rate (**e**) or time to peak metabolism (**f**) for treated samples compared to control for three replicates in each condition. Statistical significance was calculated using linear mixed-effects models. Annotations represent a significant effect of aggregation, based on pairwise t-test using Satterthwaite’s method with the lmerTest R package (*P*<0.001=***; *P*<0.01=**; *P*<0.05=*; *P*<0.1=(+, marginal significance).

To investigate whether non-surfaced-attached aggregates and single cells are the source of the differentially susceptible subpopulations observed in the previous experiments, we first repeated antibiotic treatments, where aggregates and single cells were separated by filtration (Fig. 5a). Using a linear, mixed-effects model, we identified that colistin treatment was ~1.7 fold more efficacious than tobramycin, while the combination treatment was 4.4-fold higher. There was no significant difference in antibiotic efficacy due to aggregation status, though colistin sulphate displayed a marginal significance (*P*=0.051), compared to no significance for colistimethate sodium. Of the overall variability in antibiotic efficacy, 40.5% of the observed variance was explained by treatment and only 5.3% was explained by aggregation status.

We then examined a hyper-aggregating *P. aeruginosa* strain (ΔwspF), which would quickly generate aggregates, allowing examination of aggregates during exponential growth. From an exponentially growing culture, we first quantified the proportion of aggregates in batch cultures of each strain using confocal microscopy. The ΔwspF mutant contained a significantly higher proportion of aggregated biomass (*P*=0.0009), with 54.8% ± 3% of the total biomass being associated with aggregates, compared to 28.5% ± 3% for the WT (Fig. 5b). The average cross-sectional area (µm^2^) for aggregates was 79.9±19 and 44.6±7 µm^2^ for ΔwspF and WT, respectively. The size of aggregates (*P*=0.07) and total biomass per drop (*P*=0.07) were slightly, but not significantly, higher in ΔwspF than WT. Increased aggregation of the ΔwspF strain was also confirmed visually (Fig. 5c).

We then hypothesized that if aggregation affected antibiotic tolerance, the ΔwspF mutant would respond differently to treatment than the WT. To test this, we utilized microcalorimetry to examine the effect of treatment on bacterial growth. Qualitatively, the effect of treatment on the microcalorimeter traces was affected both by treatment and strain (Fig. 5d). For tobramycin, WT cultures showed a dramatic suppression of metabolic activity compared to the untreated control. Treated samples exhibited a low and wide peak, maintaining a low metabolic output for up to 12 h, contrasting the sharp and high peak of the control. The ΔwspF mutant showed a similar response to tobramycin characterized by a low sustained metabolic output for up to 20 h. Compared to tobramycin, colistin had no detectable impact on the exponentially growing WT. Treated samples and untreated controls were indifferent, indicating negligible susceptibility. For ΔwspF, colistin produced a delay of metabolic activity, but the peaks had a comparable magnitude to that of the untreated control.

To quantify these qualitative observations, we compared the effects of aggregation (wspF vs WT) on maximum metabolic rate (MMR) (Fig. 5e) and time to peak metabolic rate (TTP) (Fig. 5f) during colistin and tobramycin treatment and modelled these with linear, mixed-effects models. For MMR, we observed small, but significant effects of aggregation on MMR, where aggregates demonstrated slightly lower MMRs after treatment than the WT. However, only ~5.5% of the overall variability in MMR observed was due to aggregation, while 95.5% was due to antibiotic treatment. When considering the TTP, aggregation significantly increased the TTP for tobramycin (*P*=0.007), but not colistin (*P*=0.131). Here, aggregation played a more significant role – explaining ~41.9% of the observed variance when compared to 59.1% of variance explained by treatment.

## Discussion

The previously long-held generalization that batch cultures, a common starting point for many microbiological methods, contain a homogenous population of single cells is recently becoming increasingly challenged [33]. In the current study, we provide evidence that bath cultures of *P. aeruginosa* contain multiple subpopulations – stratified both by growth phase and aggregation status. We demonstrate that the growth phase directly affects antibiotic tolerance, but the effect of aggregation is antibiotic specific. These results are aligned with current evidence that single cells, non-surface-attached aggregates and the overall growth phase of the system are essential for understanding antibiotic tolerance – which is especially relevant in the case of chronic infections.

We clearly demonstrate that antibiotic efficacy is modulated by growth phase as well as media conditions. Tobramycin worked well on fast-growing cultures but lost efficacy in older, stationary-phase cultures, while colistin was more efficacious on slow-growing cultures. The differential susceptibilities of bacteria to colistin and tobramycin treatment have been demonstrated in biofilm experiments [12], but not in batch culture. This observation brings into question the idea of whether this differential susceptibility is truly a unique consequence of stationary biofilm formation, when most cells in a batch culture are single-cells or free-floating aggregates. By treating stationary-phase cultures with tobramycin in fresh media, we were able to increase the bacteria’s metabolism and increase susceptibility, further supporting the link between tolerance and metabolism. Microscopy of these cultures also visually demonstrated that there were aggregates present. Previous experiments have also shown that increasing growth rate, such as by increasing access to oxygen, has led to increased antibiotic efficacy [36].

This raised the question – to what extent does aggregation play a role in tolerance compared to growth rate? By separating single cells and aggregates with filtration as well as utilizing a hyper-aggregating mutant, we observed that aggregation demonstrated significant effects on the outcome of antibiotic treatment. While significant, the magnitude of aggregation on log-reduction was small, compared to the effect of antibiotic treatment (tobramycin vs colistin). Given that these antibiotics have been shown to affect actively growing and stationary-phase bacteria differently, these results suggest that the differentiation between actively growing and stationary-phase is more important for determining the outcome of antibiotic treatment than aggregation. Interestingly, the effects of aggregation were more apparent on microcalorimetry results, the hyper-aggregating strain had a later time-to-peak metabolism than the WT (after tobramycin treatment) as well as lower peak metabolism (with both treatments). These results suggest that aggregates may still harbour tolerant subpopulations, which show reduced metabolism compared to single cells. This is also supported by the finding that aggregates generate less GFP in our arabinose-inducible mutant. Some limitations are important to note in this study. Most experiments in this study were conducted with colistimethate sodium, which is commonly used in the clinic and is a prodrug to colistin. Colistimethate sodium is spontaneously hydrolysed to colistin in solution, though the rate depends on temperature and media [37]. We demonstrated a slightly higher effect of colistin sulphate on aggregates after 24 h, suggesting that the efficacy of colistimethate sodium may underestimate the direct effect of colistin. The concentrations of antibiotics were determined based on levels of tobramycin and colistin observed in cystic fibrosis sputum and thus, much higher than would be utilized during, for example, intravenous treatment [38,39]. Additionally, potential confounding factors should be considered regarding our gfp-reporter strain. This strain utilizes an arabinose-inducible GFP reporter to measure metabolic activity, which is dependent on the ability of arabinose to diffuse. Biofilm matrix components may inhibit this diffusion, reducing the overall expression of GFP in the aggregates. Further studies may benefit from utilizing a ribosomal promoter of the unstable GFP, which would provide a more direct quantification of growth via expression of ribosomes.

The implications of these findings are relevant not only for *in vitro* models of bacterial growth but also for a better understanding of bacterial growth *in vivo*. *In vitro*, it is important for standardized bacterial growth methods to account for the fact that the inoculum may not represent a homogenous population. For example, it has been shown that inoculation methods causing the formation of more aggregates affected antibiotic tolerance experiments in batch cultures [22], but other experiments show that biofilms formed by aggregates or single cells did not show differential susceptibility [40]. Thus, the effects of metabolic and tolerant subpopulations likely depend on the *in vitro* model used, and the contribution of heterogeneous subpopulations may have a larger impact in liquid batch cultures than in *in vitro* biofilm growth models. This, however, may just as well be model or species-dependent. *In vivo*, the establishment of subpopulations and formation of non-surface-attached aggregates is a topic of continued discussion. Recent reviews highlight the formation of microenvironments and non-surface-attached aggregates and their influence on bacterial physiology [15,41]. Furthermore, both non-surface-attached aggregates and single cells are common during infections [42]. While our study demonstrated that non-surface-attached aggregates represented a subpopulation of slower-growing organisms in liquid batch cultures *in vitro*, it is important to note that bacterial growth *in vitro* drastically deviates from the exaggerated differences observed *in vivo*. We showed only a small difference in growth between single cells and aggregates in this study, and it is likely that this effect is not directly translatable to *in vivo* infection.

## Conclusion

In conclusion, we demonstrate that metabolic distinct and antibiotic-tolerance-associated subpopulations exist in shaken batch cultures of *P. aeruginosa,* just as in flow cell biofilms. These subpopulations are stratified by both aggregation and growth rate, each of which affects tolerance to antibiotics. Our results suggest that growth rate (and antibiotics targeting these metabolic groups) plays a more significant role than aggregation on the outcome of antibiotic treatment. This is especially relevant to chronic bacterial infections, where biofilm and non-surface-attached aggregates are often implicated. Future research may advance the focus on determining the origin of metabolic subpopulations, both aggregate-associated and in single cells, and how these affect antibiotic tolerance.

## Abbreviations

EMM: estimated marginal means
IMC: sothermal microcalorimeter
LB: lysogeny broth
LD: log density
LR: log reduction
MLD: mean log density
MMR: maximum metabolic rate
TTP: time to peak metabolic rate
VBNC: viable-but-non-culturable cell
